# Low-Voltage and Low-Power True-Single-Phase 16-Transistor Flip-Flop Design

**DOI:** 10.3390/s22155696

**Published:** 2022-07-29

**Authors:** Jin-Fa Lin, Zheng-Jie Hong, Jun-Ting Wu, Xin-You Tung, Cheng-Hsueh Yang, Yu-Cheng Yen

**Affiliations:** 1 Department of Information and Communication Engineering, Chaoyang University of Technology, Wufeng District, Taichung City 413310, Taiwan; s10730093@gm.cyut.edu.tw (J.-T.W.); s11030617@gm.cyut.edu.tw (X.-Y.T.); s10830106@gm.cyut.edu.tw (C.-H.Y.); s10830053@gm.cyut.edu.tw (Y.-C.Y.); 2 Department of Computer Science and Engineering, National Chung Hsing University, Taichung 40227, Taiwan; zjhong@smail.nchu.edu.tw

**Keywords:** low voltage, low power, flip-flop, pass transistor logic

## Abstract

A low-voltage and low-power true single-phase flip-flop that minimum the total transistor count by using the pass transistor logic circuit scheme is proposed in this paper. Optimization measures lead to a new flip-flop design with better various performances such as speed, power, energy, and layout area. Based on post-layout simulation results using the TSMC CMOS 180 nm and 90 nm technologies, the proposed design achieves the conventional transmission-gate-based flip-flop design with a 53.6% reduction in power consumption and a 63.2% reduction in energy, with 12.5% input data switching activity. In order to further the performance parameters of the proposed design, a shift-register design has been realized. Experimental measurements at 0.5 V/0.5 MHz show that this proposed design reduces power consumption by 47.3% while achieving a layout area reduction of 30.5% compared to the conventional design.

## 1. Introduction

With the rapid development of the Internet of Things (IoT), there is an increasing demand for low-voltage and low-power SoC chips. Speed performance is no longer the main subject of the research effort; instead, layout area and power dissipation as well as leakage power consumption are the focus of the new designs [1].

A flip-flop (FF) is the basic logic component used in digital VLSI, which is densely pipelined and employs many FF-based blocks [2,3]. For example, in low-power 32-bit microcontrollers for Internet of Things applications (IoT) with minimal memory specifications, when they are synthesized from standard cells. In this design, FF will occupy more than 30% of the layout area and power consumption [4,5]. Therefore, FF is critical to the power consumption of the design and has a significant impact on chip area. With the development of new process technology, the design method of FF continues to develop. Specific application requirements such as low voltage, low power, low cost or high performance also require new designs [6,7,8,9,10,11,12,13,14,15,16,17,18,19,20,21,22,23,24]. In this work, the FF design goal is a low voltage and low power consumption with a compact layout area design solution.

**Figure 1:**
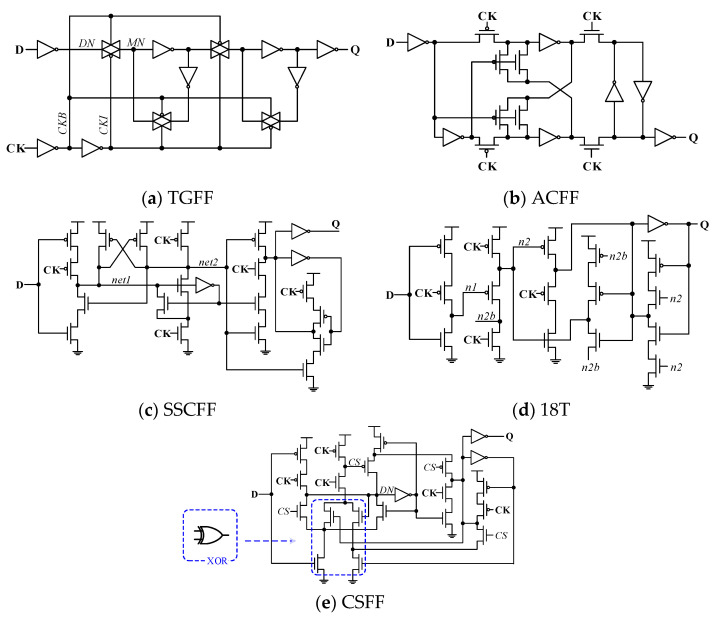
Conventional flip-flop designs. (**a**) Conventional transmission gate-based FF (TGFF). (**b**) Adaptive coupling FF (ACFF). (**c**) Static contention free single-phase-clocked FF (SSCFF), and (**d**) 18-transistor FF (18T). (**e**) Change sensing FF (CSFF).

A conventional transmission gate (TG)-logic-style-based design named TGFF is widely used in the field of digital systems today. The major problem of this design is the excessive loading on the clock signal (a total of 12 transistors driven by the clock signal), as shown in Figure 1a. As a result, there is considerable dynamic power consumption, even when the input data signal switching activity is zero. Recently, true single-phase clocking (TSPC)-based FF designs are presented for low voltage and low power applications. The key idea is reducing the loading capacitance of clock signal by using both logic and circuit level simplification.

Figure 1b. shows the adaptive coupling flip-flop (ACFF) [6]. Unlike the conventional TGFF, this FF uses a differential latch circuit with pass transistor logic (p-type for master latch and n-type for slave latch) for the true-single-phase clocking operation. In order to reduce the circuit effects of process variation on the master latch circuit of FF design, a pair of level restore circuits was employed into the cross-coupled inverter. In this low-power FF design, the clock signal drives only 4 (2 pMOS transistor and 2 nMOS transistor) for a total of 22 transistors. When this FF design is operating at lower input data switching activity, the very light clock loading can significantly reduce dynamic power. However, latch circuit design based on p-type pass transistor logic result in longer setup time performance, even with the addition of a level recovery circuit. Moreover, the data contention issue in the slave latch circuit deteriorates as the switching activity increases, and thereby reducing the power saving advantage. As a result, ACFF design is not suitable for low supply voltage operation [11].

Figure 1c shows the schematic of static contention free true-single-phase-clocked (TSPC) flip-flop, named SSCFF, designed to reduce these circuit problems [8]. It consists of a traditional dynamic circuit structure TSPC flip-flop design with 9-transistor [15] and additional transistors to ensure its static operation. This FF design can better provide both power and energy consumption compared with conventional designs. It also overcomes the problem that previous ACFF designs cannot operate at low operating voltages. However, the height of its pull-down circuit adds up to three. This requires large-sized nMOS transistors and results in a bigger layout area. Another retentive TSPC-based FF design is presented [13] as shown in Figure 1d. It is simplified from the SSCFF design and uses only 18 transistors. Because the MOS circuit is too simplified, the output drive capability is lost. Not only that, due to the temporary short-circuit paths in FF design, this also affects the robustness of the circuit operation, especially at low supply voltage.

The CSFF (change sensing flip-flop) design includes an XOR logic in its master stage to check both input data with output Q as shown in Figure 1e [18,19]. In this FF design, the input data are captured only when the discrepancy occurs. This gives a performance edge in power over the conventional TGFF design when the switching activity is lower. However, some internal floating nodes already exist, and use up to 24 transistors [20,21].

In this paper, a novel low-voltage and low-power FF design using pass transistor logic circuit scheme with only 16 transistors is presented. It also follows the circuit behavior of TSPC-based FF design to reduce loading of clock signal. The design exhibits the lowest layout area and better various performances while compared with previous FFs.

The rest of this paper is organized as follows. In Section II, the same low power FF designs are first reviewed, and the proposed design is then described in detail. The simulation settings and design performance indices are described in Section III. Detailed post-layout simulation results, chip measurement results and comparisons with previous FF designs are showed next. The conclusions are presented in Section IV.

## 2. Proposed Low Power 16-Transistor FF Design

**Figure 2:**
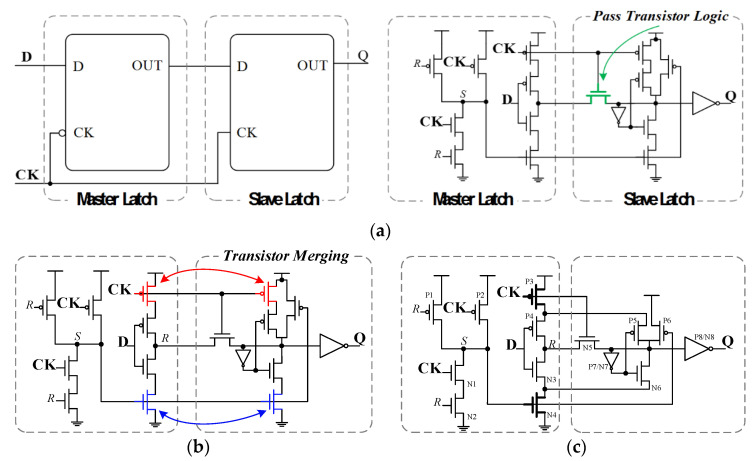
Design evolution of the proposed 16-transistor FF design from pass transistor logic scheme with MOS circuit compression. (**a**) Structure of master–slave-based FF using CMOS logic style multiplexer and pass-transistor-logic-based latch. (**b**) MOS Transistor-level schematic (merged scheme). (**c**) The proposed 16T FF design.

Based on these shortcomings of previous low-power FF designs, we refer to the design of both ACFF and 18T, and propose an enhanced FF design to alleviate the above circuit problems. Referring to Figure 2, the proposed FF design adopts different circuit structure, i.e., a modified TSPC-based latch design at master stage and a pass-transistor-logic-based latch design at the slave stage. The proposed circuit design contains 18 transistors, as shown in Figure 2a. Referring to this schematic, at the transistor level, we use the following steps to further reduce the number of transistors. In terms of pull-down circuits, one signal “S” that controls the nMOS transistor can be merged by the two pull-down paths, as shown in Figure 2b (color in blue). Additionally, on the pull-up circuit side, a signal “CK” that controls the pMOS transistor can be merged by the two pull-up paths, as shown in Figure 2b (color in red). Therefore, a total of two redundant MOS transistors have been successfully removed. This includes a pMOS transistor deactivated by clock signal. This simple circuit scheme not only reduces power performance and circuit complexity, but also reduces the capacitive loading of the clock signal. The proposed FF design has a total transistor count of 16 and only 4 clock transistors, whereas the conventional TGFF design has a transistor count of 24 with 12 clock transistors, which ensures its low power behavior, as shown in Figure 2c.

In the proposed design, the setup time parameter is determined by the delay from input D to node *S*. Compared with the ACFF, SSCFF and CSFF designs, a smaller delay can be achieved when operating in the transparent case, resulting in a better setup time performance of the design. Since the proposed design also uses the pass logic transistor scheme to reduce circuit complexity, both the loose threshold voltage and weak drive capability problems will have to be considered. When the input signal clock is high and data are low (CK = 1, D = 0), the node *R* of the proposed FF design will charge to high, i.e., node *S* discharge to low. In this case, pMOS transistor P6 turns on and charges the node QB to high, as shown in Figure 2c. Therefore, the above circuit problems can be ignored. In other words, our design can successfully achieve circuit complexity reduction and timing parameter enhancement simultaneously.

**Figure 3:**
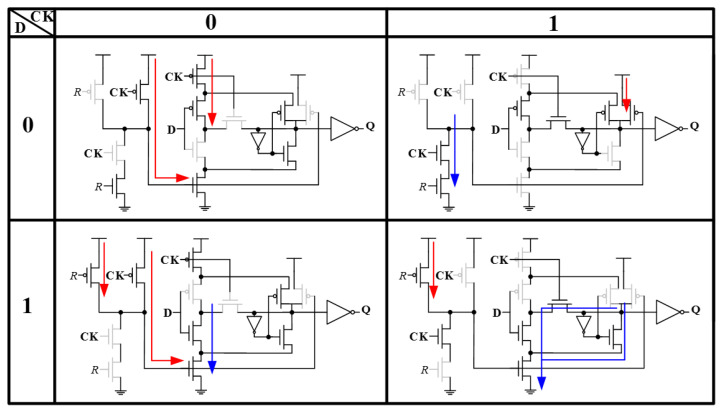
Operation diagram of the proposed FF design at different inputs (data and clock).

Figure 3 shows the detail circuit operation of the proposed FF design at different input data and clock cases. No data contention paths were observed under any circumstances. When the input clock (CK) is low, devices on input data (D) only change the value of the node *R* in the master latch. The pass transistor logic scheme employed in the slave latch remains isolated from input data when the clock is low; changing the stage on node *R* will not cause any data corruption from the slave circuit. When clock is high, input data will isolate, and output the previously latched data in the master circuit.

**Figure 4:**
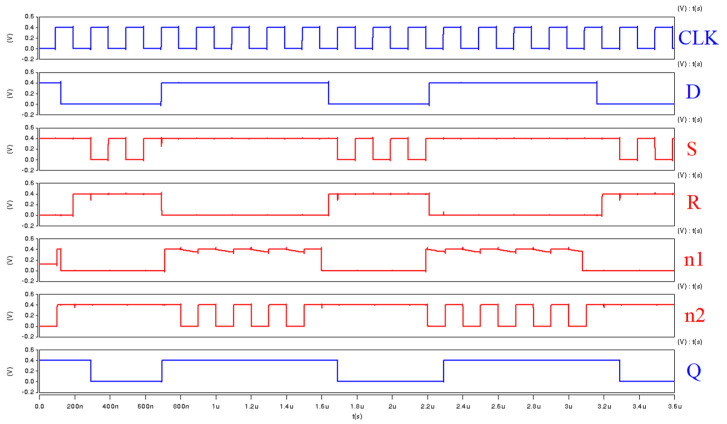
Snapshots of simulation waveforms, the proposed design is the top row come next is 18T design.

Figure 4 shows the simulation waveforms of both the proposed FF design and the 18T design at the supply voltage of 0.4 V. The proposed FF design works correctly at near-threshold supply voltage. It can also be seen from the simulation waveforms that node n1 floats in the 18T design. Thus, the 18T design is not suitable for low supply voltage applications. In conclusion, our design achieved both power consumption enhancement and MOS circuit reduction, even when operating at low supply voltage.

## 3. Simulation Results

In order to show the performance of the proposed FF design, TGFF, ACFF [6], SSCFF [8] 18T [13], and CSFF [19] designs (as shown in Figure 1), have been realized based on TSMC-180 nm and TSMC-90 nm [22,23]. The size of the MOS transistor depends on the optimization of the power–delay product (PDP_CQ_) and the function at lower supply voltage. The MOS transistor sizes with minimum length of the proposed design are shown in Table 1 and Table 2, respectively. It is important to note that in the ACFF design, the width of the p-type pass transistor must be enlarged to provide better setup time to ensure its current operation. However, the ACFF design still cannot operate in the SF corner [24,25]. All input signals (clock and data) are generated after passing through the buffer circuit to account for the effects of rise time and fall time delays.

**Figure 5:**
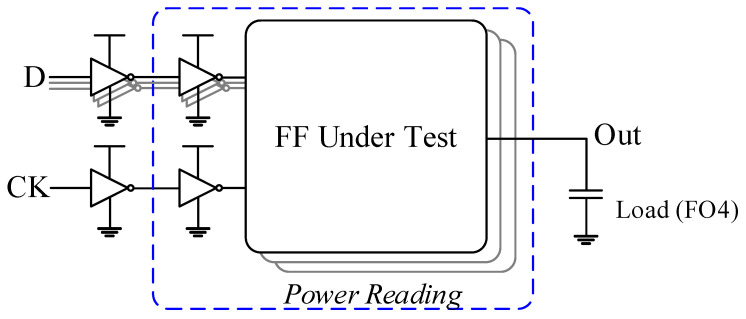
Test bench for flip-flop design simulations.

The operating condition used in post-layout simulations is 0.5 MHz/0.5 V for TSMC-180 nm and 5 MHz/0.4 V for TSMC-90 nm, respectively. A total of five test patterns were used in the simulation, each pattern exhibiting a different activity. The input data toggle the active range from 100% to 0% [26,27,28,29]. The model setup for the design simulation in this paper is shown in Figure 5. Note that it has a clock buffer driving 4 FFs in order to simulate a realistic scene. The circuit’s current driving the FF design was measured and divided by 4. Therefore, the average power consumption performance measured in this work also takes into account the power consumption of clock signal driven. The simulation results including transistor count, layout area, timing parameter (hold time, setup time, CQ delay and DQ delay), average power consumption performance, and power–delay product, i.e., energy consumption performance, are summarized in Table 3 and Table 4.

**Figure 6:**
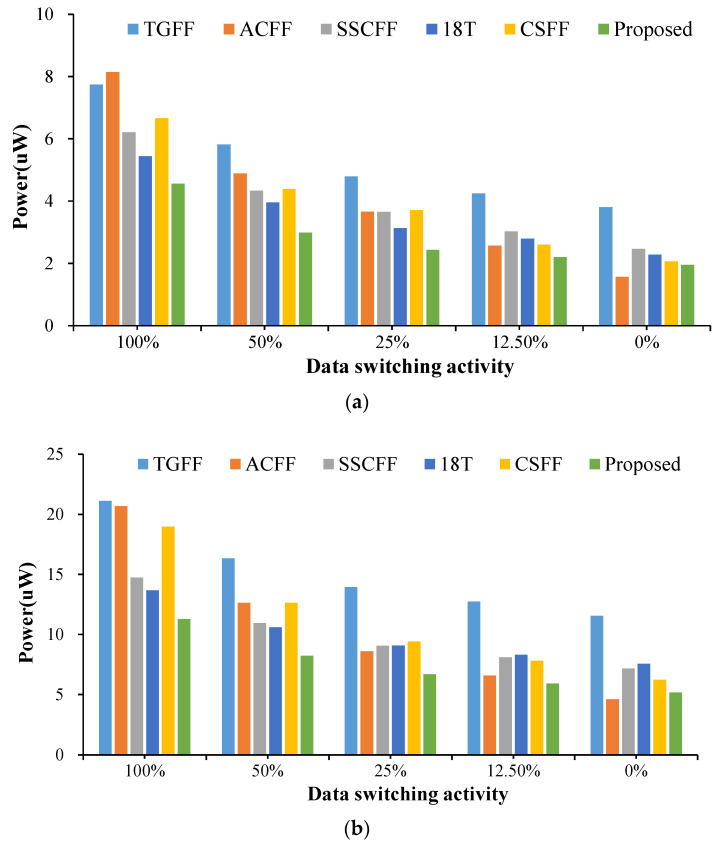
Average power consumption of FF designs @different data switching activity at the CMOS technology of (**a**) TSMC-180 nm and (**b**) TSMC-90 nm.

In terms of total transistor count, the proposed FF design uses the fewest total transistor counts and has the smallest area among these FF designs. As for the average power consumption performance as shown in both Table 3 (TSMC-180 nm) and Table 4 (TSMC-90 nm), except for one case, the proposed FF design is the most power efficient (when the input data toggle activity is 0%, our design is slightly higher to the ACFF). Compared with the TGFF design, our design can reduce power consumption achieving from 42.38% to 49.17% (180 nm) and 46.64% to 55.36% (90 nm), respectively. For 12.5% of input data switching activity, the average power performance reductions for TGFF, ACFF, SSCFF, 18T and CSFF were 47.88%, 14.01%, 27.06%, 20.79%, 15.0% (180 nm) and 53.57%, 10.03%, 26.91%, 28.85%, 24.10% (90 nm), respectively. The detailed average power consumption results are also recorded in Figure 6. Note that, in CSFF design, a lighter loading of the clock in addition to the charge sensing circuit scheme of the FF design can reduce the power at lower switching activity. However, the extra circuit (change sensing) in the master latch of the design deteriorates as the switching activity increases, and the performance edge of the power consumption is thus diminished.

**Figure 7:**
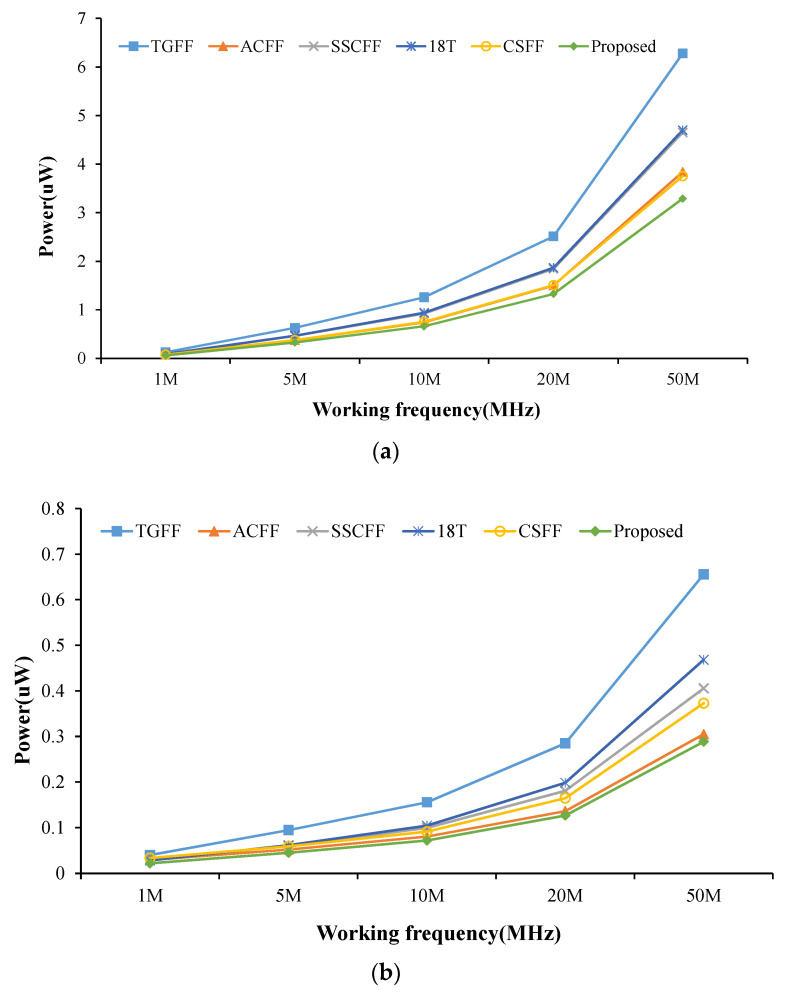
Average power of FF designs @different operation frequency at the CMOS technology of (**a**) TSMC-180 nm and (**b**) TSMC-90 nm.

**Figure 8:**
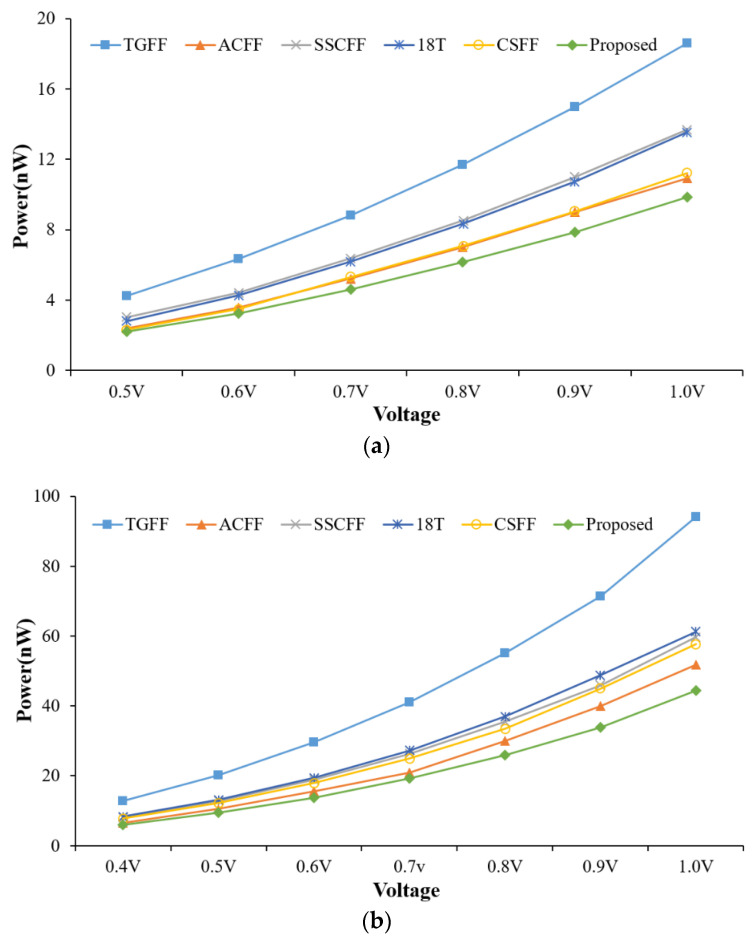
Average power of FF designs @different supply voltage at the CMOS technology of (**a**) TSMC-180 nm and (**b**) TSMC-90 nm.

Figure 7 shows the comparison of average power performance at different operating frequencies. The supply voltage V_DD_ is setting 1.8 V for 180 nm and 1.0 V for 90 nm, respectively. Our design is the best power efficiency in all cases. The conventional TGFF design has the worst power consumption due to it having a larger clock loading (total 12-transistor driving by the clock signal) than other low power FF designs. Figure 8 shows the comparison of average power consumption at different supply voltages. The operation frequency is 0.5 MHz for 180 nm and 5 MHz for 90 nm, respectively. Unsurprisingly, the proposed FF design also has the best power performance in all cases.

**Figure 9:**
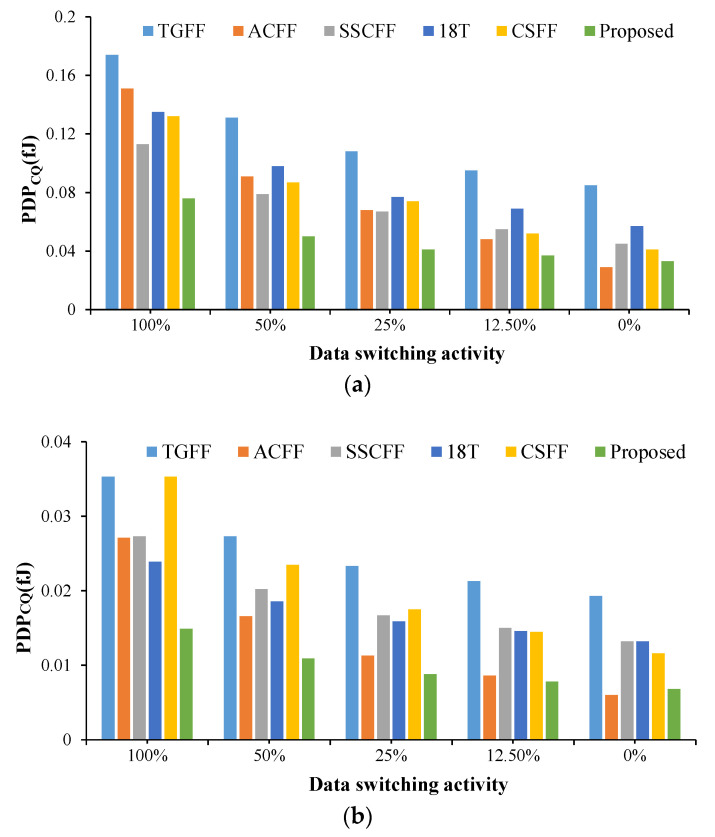
Power–delay product of FF designs @different input data switching activity at the CMOS technology of (**a**) TSMC-180 nm and (**b**) TSMC-90 nm.

In addition to average power consumption performance, this paper uses the PDP_CQ_ index as a comprehensive performance index, as shown in Figure 9. When the input data switching activity is 12.5%, the PDP_CQ_ of the proposed FF design is 22.76~61.30% smaller than that of the compared FF designs.

**Figure 10:**
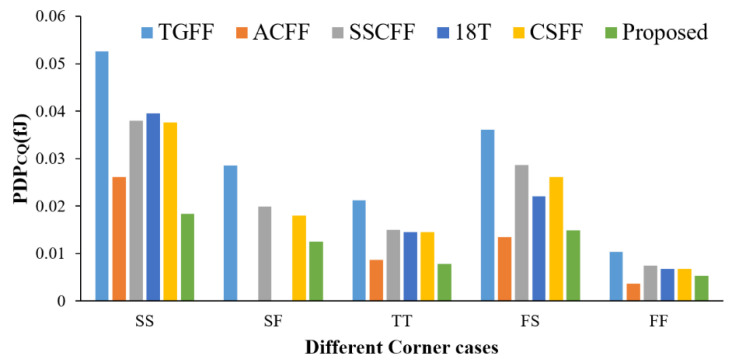
PDP of FF designs @different process corner.

Figure 10 shows the PDP performance versus process variation at 12.5% switching activity (TSMC-90 nm only). For each process corner, scan the setup time value to obtain the best PDP_CQ_ performance. Compared with other FF designs, our design has better PDP_CQ_ performance in all cases. This is a testament to the consistent performance benefits of the proposed design. Notably, the conventional TGFF design has the worst performances. As mentioned before, since the ACFF design uses a single p-type pass transistor logic style latch in master stage and data contention problem in the slave stage, it does not operate correctly at the SF corner. Additionally, in the design of 18T, because of the circuit problems mentioned above, it also cannot operate correctly in the SF corner.

In tern of timing parameters of the FFs, Table 1 and Table 2 lists the setup time, the hold time, the Clock-to-Q (CQ) delay and the Data-to-Q (DQ) delay of these FFs. As explained before, the setup time for ACFF is much greater than for other FF designs. Compared with the ACFF design, the proposed design is also implemented by using pass transistor logic. However, the Clock-to-Q delay and setup time are significantly improved due to the successful elimination of the threshold voltage loose problem while simplifying the circuit complexity.

In terms of hold time parameters, ACFF and TGFF have negative hold times, whereas TSPC-based FF designs, i.e., SSCFF, 18T, CSFF and the proposed FF design, have positive values. The hold time for these TSPC-based FF designs must be positive to ensure that the input data transition from the slave latch is complete. The proposed design also results in better CQ delay performance. Latency is 8.6% shorter than the closest TSPC-based competitor, i.e., SSCFF design.

**Figure 11:**
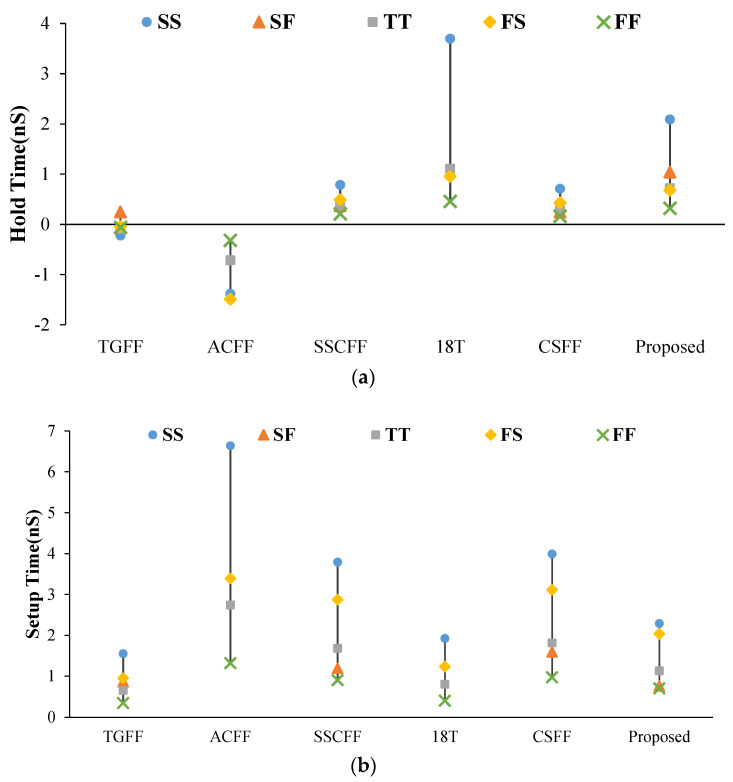
Timing variation simulation for different process corners. (**a**) Hold time. (**b**) Setup time.

Figure 11 shows the variations in setup time and hold time performance affected by process variation (TSMC-90 nm). Because of the single-type pass-logic-style-based latch design, the setup time performance of the ACFF design were significantly higher than other FF designs. Although our design also uses pass-transistor-logic-structure-minimization circuit schemes, its setup time and hold time variation are well controlled.

**Figure 12:**
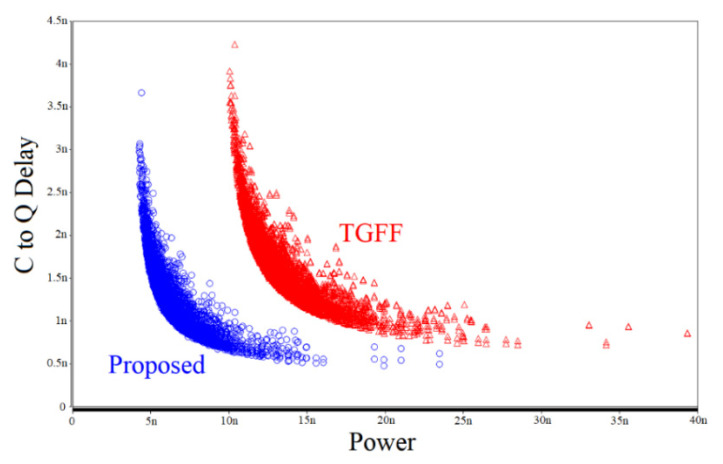
Monte Carlo simulation results.

Figure 12 shows the Monte Carlo simulation results of power–delay product (PDP_CQ_) derived by executing 3000 runs. Two flip-flop designs are simulated: the traditional TGFF design and the proposed FF design. The graph has a format of average power performance as the *x*-axis and the delay performance (Clock to Q delay) as the *y*-axis. Thus, the closer the point is to the lower left part of the drawing, the better the performance of the FF design. From the results, the advantages of our design in the simulation experiments are obvious.

**Figure 13:**
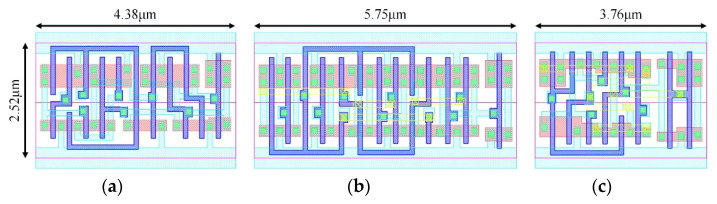
Layout of proposed design and TGFF design (TSMC 90 nm). (**a**) 18T; (**b**) TGFF; (**c**) Proposed.

Figure 13 shows the layout schematics of 18T, TGFF and the proposed FF designs. All FF designs have a fixed height of 2.52 μm and layers up to metal-2 are employed when drawing the layout. The layout area size of our design is only 3.76 μm by 2.52 μm. The layout area can saved up to 34.61% space compared with the conventional TGFF design. It is important to note that although the ACFF design has fewer transistors than conventional TGFF design, in order to ensure that the operation requirements of lower supply voltage operation are met, a larger transistor size is required to increase the layout area. Finally, the area size of the TSPC-based SSCFF design is 6.26% larger than that of the conventional TGFF design for the same number of transistors (24), since the SSCFF requires larger transistors and a complex layout structure.

**Figure 14:**
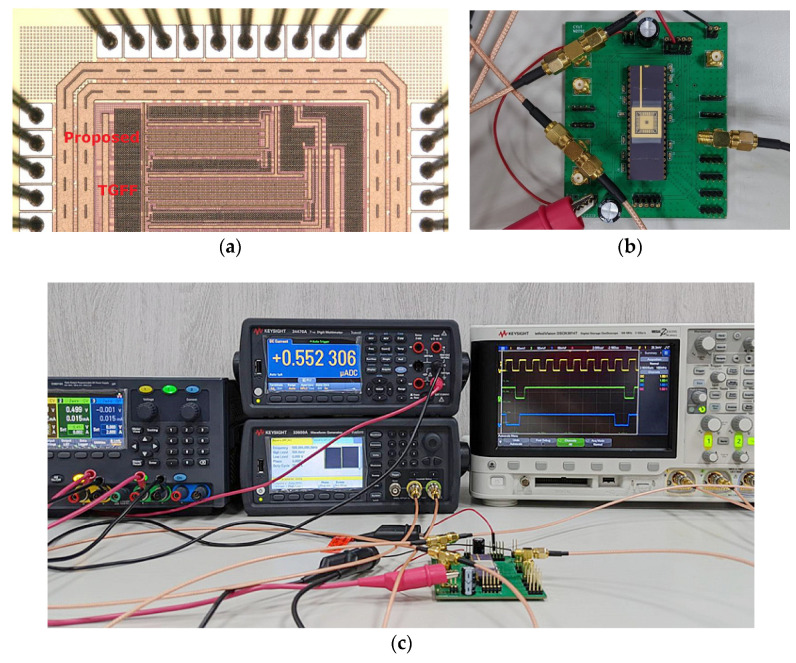
Chip Implementation and Measurement Results. (**a**) Die photograph. (**b**) Testing PCB with a fabricated chip. (**c**) Test platform.

The conventional TGFF design and the proposed FF design further extended to a 256-bit shift register design [30]. The die photographic of fabricated chip and PCB are shown in Figure 14a,b. The test platform used in this work is shown in Figure 14c and combines a digit multi-meter (KEYSIGHT-34470A), a digital storage oscilloscope (KEYSIGHT-DSOX3014T), a triple output programmable DC power supply (KEYSIGHT-E36313A) and an arbitrary waveform generator (KEYSIGHT 33600A).

**Figure 15:**
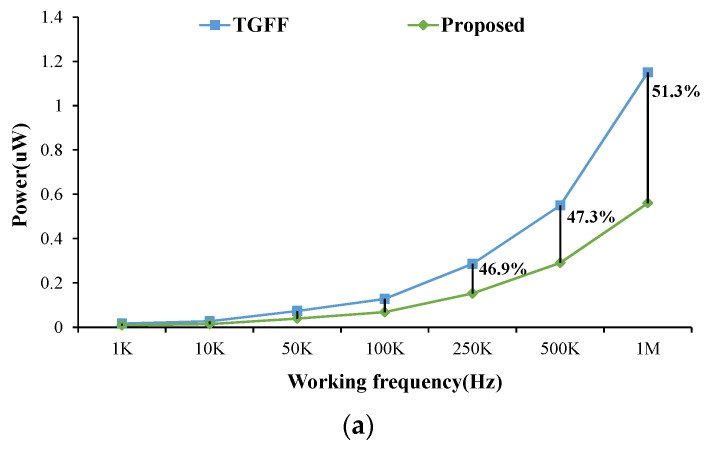

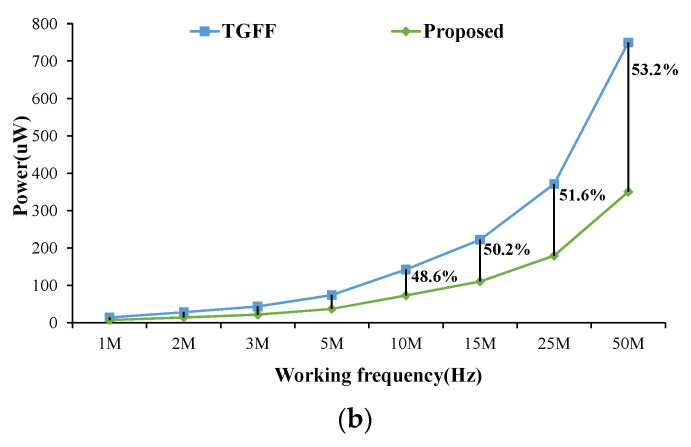
Measured power consumption of 256-bit shift-register design with different operation frequencies at (**a**) 0.5 V and (**b**) 1.8V.

Figure 15 shows the measured power consumption (including clock buffer) at different operation frequencies. The supply voltages are set as 0.5 V and 1.8 V, respectively. From the results, it can be seen that our design reduces over 47% power at 0.5 MHz, and the power benefit is maintained as frequency is increased. Table 5 shows the measurement comparison table between the TGFF design and the proposed design. Due to its circuit novelty, our design reduces the power consumption by more than 53%, whereas the layout area is reduced by 30.5%. Finally, for the design running at 32768Hz, the required supply voltage of our design can be further reduced to 0.293V, and the total power dissipation is only 31 nW. All the above results show that the proposed FF design has lower power and energy performance and a smaller chip area than previous FFs. Therefore, the proposed FF design is suitable for low voltage and low power system chips.

## 4. Conclusions

A novel low-power flip-flop design with compact layout area is presented in this paper. This FF design uses a master–slave structure and combines a pass-transistor logic circuit scheme for low-power applications. A comprehensive evaluation shows that out of all of the compared FF designs, the proposed design is the most economical in terms of both energy and power consumption. Due to the novelty of the circuit we designed, it also requires minimal chip area, making it a viable option for low-power and low-cost applications.

## Figures and Tables

**Table 1:** Transistor size of proposed FF design (180 nm).

Name	W(nm)	Name	W(nm)	Name	W(nm)	Name	W(nm)
P1	250	P5	500	N1	1000	N5	500
P2	250	P6	500	N2	1000	N6	500
P3	1500	P7	250	N3	250	N7	250
P4	1000	P8	1000	N4	500	N8	500

**Table 2:** Transistor size of proposed FF design (90 nm).

Name	W (nm)	Name	W (nm)	Name	W (nm)	Name	W (nm)
P1	200	P5	300	N1	450	N5	300
P2	200	P6	300	N2	450	N6	200
P3	600	P7	200	N3	200	N7	200
P4	400	P8	600	N4	300	N8	300

**Table 3:** Comparison summary of various FFs @0.5 MHz/0.5 V/TT-Corner (180 nm).

FF Designs	TGFF	ACFF ^1^	SSCFF	18T ^1^	CSFF	Proposed
Transistors CK/Total	12/24	4/22	5/24	4/18	5/24	4/16
Cell Width(um)	10.86	13.52	11.84	8.81	13.37	7.52
Setup Time(nS)	10.95	37.48	19.68	10.55	21.58	11.76
Hold Time (nS)	−3.10	−8.59	1.41	12.15	1.04	7.77
CQ Delay(nS)	22.44	18.55	18.23	24.76	19.85	16.66
DQ Delay(nS)	33.39	56.03	37.91	35.31	41.43	28.42
Power@100% (nW)	7.74	8.15	6.21	5.44	6.66	4.46
Power@50% (nW)	5.82	4.89	4.34	3.96	4.39	2.99
Power@25% (nW)	4.80	3.67	3.66	3.13	3.71	2.44
Power@12.5% (nW)	4.24	2.57	3.03	2.79	2.60	2.21
Power@0% (nW)	3.80	1.56	2.47	2.28	2.07	1.95
Power–Delay Product@12.5% (aJ)	95.15	47.67	55.24	69.08	51.61	36.82

^1^ SF corner failure when at supply voltage of 0.5 V.

**Table 4:** Comparison summary of various FFs @5 MHz/0.4 V/TT-Corner (90 nm).

FF Designs	TGFF	ACFF ^1^	SSCFF	18T	CSFF	Proposed
Cell Width(um)	5.75	6.23	6.11	4.38	6.83	3.76
Setup Time(nS)	0.65	2.74	1.68	0.8	1.81	1.13
Hold Time (nS)	−0.15	−0.83	0.22	1.0	0.3	0.6
CQ Delay(nS)	1.67	1.31	1.85	1.75	1.86	1.32
DQ Delay(nS)	2.32	4.05	3.53	2.55	3.67	2.45
Power@100% (nW)	21.12	20.68	14.73	13.67	18.97	11.27
Power@50% (nW)	16.34	12.64	10.94	10.61	12.62	8.22
Power@25% (nW)	13.95	8.60	9.05	9.09	9.40	6.69
Power@12.5% (nW)	12.75	6.58	8.10	8.32	7.80	5.92
Power@0% (nW)	11.56	4.61	7.16	7.56	6.22	5.16
Power–Delay Product@12.5% (aJ)	21.29	8.62	14.99	14.56	14.50	7.84

^1^ SF corner failure when at supply voltage of 0.4 V.

**Table 5:** Chip simulation and measured results @0.5 V/0.5 MHz.

256-Bit Shift Registers	TGFF	Proposed
Layout Area (um^2^)	15,979.7	11,104.7
Average Power consumption (uW)-Simulation	0.49	0.27
Average Power Consumption (uW)-Measured	0.55	0.29

## Data Availability

The data presented in this study are available on request from the corresponding author.
